# Interactions of Attenuated *Mycobacterium tuberculosis phoP* Mutant with Human Macrophages

**DOI:** 10.1371/journal.pone.0012978

**Published:** 2010-09-24

**Authors:** Nadia L. Ferrer, Ana B. Gomez, Olivier Neyrolles, Brigitte Gicquel, Carlos Martin

**Affiliations:** 1 Grupo de Genética de Micobacterias, Departamento de Microbiología, Medicina Preventiva y Salud Pública, Universidad de Zaragoza, Zaragoza, Spain; 2 Unit of Mycobacterial Genetics, Institut Pasteur, Paris, France; 3 Centro de Investigación Biomédica en Red (CIBER) Enfermedades Respiratorias, Instituto de Salud Calos III, Madrid, Spain; University of Delhi, India

## Abstract

**Background:**

*Mycobacterium tuberculosis phoP* mutant SO2 derived from a clinical isolate was shown to be attenuated in mouse bone marrow-derived macrophages and *in vivo* mouse infection model and has demonstrated a high potential as attenuated vaccine candidate against tuberculosis.

**Methodology/Principal Findings:**

In this study, we analyze the adhesion and the intracellular growth and trafficking of SO2 in human macrophages. Our results indicate an enhanced adhesion to phagocitic cells and impaired intracellular replication of SO2 in both monocyte-derived macrophages and human cell line THP-1 in comparison with the wild type strain, consistent with murine model. Intracellular trafficking analysis in human THP-1 cells suggest that attenuation of SO2 within macrophages could be due to an impaired ability to block phagosome-lysosome fusion compared with the parental *M. tuberculosis* strain. No differences were found between SO2 and the wild-type strains in the release and mycobacterial susceptibility to nitric oxide (NO) produced by infected macrophages.

**Conclusions/Significance:**

SO2 has enhanced ability to bind human macrophages and differs in intracellular trafficking as to wild-type *M. tuberculosis*. The altered lipid profile expression of the *phoP* mutant SO2 and its inability to secrete ESAT-6 is discussed.

## Introduction

Tuberculosis (TB) is a leading cause of mortality by infectious disease throughout the world [1], along with AIDS and malaria [2], [3]. It is estimated that the bacilli of tuberculosis infect approximately one-third of the world's population causing two million deaths each year [4]. The HIV/AIDS pandemic, the deterioration of public health infrastructures in developing countries, and the emergence of multidrug-resistant (MDR-TB) and even extensively drug resistant (XDR) forms of *Mycobacterim tuberculosis* (Mtb) have further contributed to spread and transmission of the disease [5].

Currently, the *Mycobacterium bovis* bacille Calmette–Guérin (BCG) strain is the only vaccine available for the prevention of TB in humans. This live- attenuated vaccine, originally derived by serial passages of a virulent strain of *M. bovis*, has been used to prevent TB since 1921. Although BCG protects against severe forms of childhood TB, this vaccine is ineffective in preventing adult pulmonary TB, which is the major form of the disease in endemic areas [2], [3], [6]. Recently, the genome sequences of the Mtb clinical isolate H37Rv and of the BCG substrain 1173 Pasteur, which are widely used as reference strains in many mycobacterial genetics laboratories, have been published [7]. In this context, and with the relatively poor efficacy of BCG in a number of clinical trials [6], major efforts are being undertaken worldwide to develop more effective vaccines against pulmonary TB. A combination of drug treatment regimens with efficacious prophylactic vaccines could therefore potentially go a long way to reduce incidence of the disease in TB endemic areas.

The aims of the ‘classical’ live vaccine strategy are to generate host immune responses that mimic natural infection, but without causing disease [8]. The development of rationally attenuated mutants of Mtb offers the prospect of novel potential vaccine candidates against TB. By inactivating key genes involved in virulence, it allows for the possibility of using a live-attenuated vaccine that is safe and can induce the appropriate protective immune responses without generating the pathology associated with progression of disease resulting in improved protective efficacy. An advantage of the Mtb live-attenuated vaccine strategy is that many immunologically important genes are conserved in the genome, unlike *M. bovis* BCG substrains where these are deleted [9].

Two-component regulatory signal transduction systems (TCS) are important elements in the adaptive response of prokaryotes to a variety of environmental stimuli [10] and are also implicated in virulence regulation [11]. PhoP/R has been described in Mtb as a two-component regulatory system and has been shown to play an essential role in Mtb virulence [12]. In previous work, our laboratory constructed a *phoP* mutant from a clinical isolate of Mtb by introducing a marked disruption in the *phoP* gene, generating the SO2 strain which exhibited impaired multiplication *in vitro* in mouse bone marrow-derived macrophages, and *in vivo* in a mouse infection model [12].

After *in vitro* murine studies, SO2 was shown to be more attenuated than BCG in severe combined immunodeficiency (SCID) mice and to confer equivalent immunity against pulmonary TB in mice and better protection in guinea pigs when compared with BCG [13], [14]. Furthermore, recent protective efficacy studies in rhesus macaques demonstrated that SO2 vaccination significantly reduced pathology upon intratracheal Mtb infectious challenge, and provided significant protective effect by reduction in C-reactive protein levels, and body weight loss and decrease of several markers of inflammatory infection [15].

In addition to these studies, molecular assays have been performed, showing that *phoP* is involved in the regulation of complex mycobacterial cell-wall lipids implicated in the virulence of Mtb [16]–[18]. Likewise, it has been demonstrated that avirulent H37Ra strain contains a single mutation in *phoP* that accounts for the absence of complex lipids [19] and impaired ESAT-6 secretion [20] contributing to the avirulence of this strain [21].

Herein, as mycobacterial virulence is related to the initial capacity of the microorganism to survive and grow in macrophages, we study the early infection of the vaccine candidate SO2 *in vitro* in human macrophages including peripheral blood-derived macrophages (PBMC) as primary macrophages and the THP-1 cell line, which exhibits alveolar macrophage features when stimulated with PMA, in comparison with its virulent wild-type MT103 clinical strain. We compare this model with the results observed in murine macrophages in which SO2 intracellular replication is impaired. Moreover, we studied the first interaction of SO2 strain with macrophages through adhesion assays and investigated a) the trafficking involved in the internalization of mycobacteria in THP-1 cells by immunofluorescence assays to see possible differences in the phagosome-lysosome fusion and b) the produced nitric oxide (NO) by infected THP-1 macrophages, as well as the mycobacterial susceptibility to it.

## Materials and Methods

### Bacterial strains and growth conditions

*M. bovis* BCG Pasteur 1173P2, Mtb clinical isolate MT103, the MT103 *phoP* mutant SO2 (kanamycin resistant), and its complemented strain (kanamycin and hygromycin resistant), SO2-pSO5 [12], were used in this study. Mycobacteria were grown at 37°C in liquid Middlebrook 7H9 medium supplemented with 0.05% Tween 80 and 10% Middlebrook albumin dextrose catalase enrichment (ADC), and when required, the medium was supplemented with 20 µg/ml of kanamycin or hygromycin. Before use, fresh mycobacterial cultures were centrifuged at 20 g for 5 min to remove bacterial clumps.

### Eukaryotic cells

Peripheral blood mononuclear cells (PBMC) from healthy subjects from Banco de Sangre Hospital Clínico Universitario Lozano Blesa University of Zaragoza, according to institutional guidelines, were isolated by density sedimentation over Ficoll-Paque PLUS (Amersham Biosciences) and to isolate monocytes, CD14+ cells were separated from total PBMCs by rosette formation with goat erythrocytes to eliminate T lymphocytes [22] and by negative selection with magnetic particles (Dynabeads CD2 (Pan T) and CD19 (Pan B), DynalBiotech) as supplier's instructions to condense CD14+ cells, as determined by flow cytometry with the anti-CD14 antibody. Finally, monocytes were plated in a 24-well plate at a density of 6×10^5^ cells/ml in a complete RPMI-1640 medium containing 1% penicillin-streptomycin, and incubated for 7 days with medium containing 1 µl/ml of M-CSF (macrophage-colony stimulating factor, R&D Systems). The human monocytic cell line THP-1 was obtained from the ECACC collection (ECACC No. 88081201) and were cultured at 37°C in a 5% CO_2_ atmosphere. THP-1 were maintained in RPMI-1640 medium (GIBCO), containing 2 mM L-glutamine and 10% fetal bovine serum. For subsequent experiments, THP-1 cell suspensions were adjusted to a concentration of 5×10^5^ cells/ml in warm RPMI supplemented, seeded in 24-well plates adding 1 ml of suspension per well, and cells were allowed to adhere and differentiate in the presence of 10 ng/ml PMA (Sigma), at 37°C in 5% CO_2_ for 48 hours.

### Macrophage infection with *M. tuberculosis*

For infection, the medium of differentiated THP-1 and blood monocytes-derived macrophages was removed and replaced with 1 ml of bacterial suspension from each strain (Mtb MT103, its *phoP* mutant SO2, and its complemented strain SO2-pSO5) in RPMI containing 10^5^ cfu/ml, to obtain a multiplicity of infection (MOI) of 1∶10, bacteria per macrophage. After 4 h at 37°C, the medium was removed and the wells were washed three times with RPMI to remove extracellular bacteria (reduction to a non significant number of extracellular bacilli), before adding 1 ml of fresh culture medium per well. On days 0 (4 h), 1, 3, 5 and 7, cells were lysed with 500 µl of sterile water with 0.1% triton X-100, and the number of viable intracellular bacteria was counted by plating serial dilutions of the lysis solution onto Middlebrook 7H10 agar supplemented with 10% OADC (oleic albumin dextrose catalase enrichment). This infection experiment was carried out in triplicate.

### Binding of mycobacteria to THP-1

The medium overlying the THP-1 monolayer was replaced with 1 ml of ice-cold tissue culture medium. Mycobacteria were added to the monolayer at MOI of 1∶1 because of the low percentage of adherent bacteria. The mycobacteria were allowed to adhere for 4 hours at 4°C, and after the incubation period each well was rinsed 3 times with 1 ml of ice-cold Phosphate Buffered Salt Solution (PBS), pH 7.4. Monolayers were subsequently treated with 0.5 ml of Triton X-100 (Sigma) in sterile water for 10 min. Non-specific adherence to the plastic did not occur at 4°C (data not shown). Adherent bacteria were quantified by plating for colony forming units (CFU) onto Middlebrook 7H10 agar.

### Intracellular trafficking within THP-1

Macrophages were seeded on glass coverslips and infected under the same conditions as the survival experiments, at MOI of 1∶1. We used heat-inactivated MT103 as negative control, by heating bacteria at 80°C for 40 min. After 48 h of infection, coverslips were fixed with 4% paraformaldehyde (PFA) in PBS at room temperature for 30 min. Finally, PFA was removed and cells were maintained in PBS at 4°C until their labeling. For LysoTracker labelling, cells were incubated at 37°C with 50 nM of LysoTracker (Molecular Probes) during 2 h, and subsequently washed, before fixing. Then, coverslips were permeabilized and incubated with a blocking solution of PBS, containing 2% FBS, 0.1% bovine serum albumin (BSA) and 1% saponin, for 30 min and incubated with primary antibodies against LAMP-1 (late phagosome-endosome marker), CD63 (phagolysosomal marker), and anti-BCG for 1 h. As primary antibodies we used monoclonal mouse anti-human LAMP-1 (Fitzgerald) diluted 1∶100, monoclonal mouse anti-human CD63 (Fitzgerald) diluted 1∶100 and polyclonal rabbit anti-BCG diluted 1∶50 kindly provided by Dr. Natalie Winter (Pasteur Institute, Paris, France). After the primary labeling, cells were washed three times with PBS-1% saponin, and incubated with fluorescent secondary antibodies in absence of light for 1 h. As secondary antibodies: Alexa Fluor 488 F(ab')_2_ fragment of goat anti-rabbit IgG (Molecular Probes) and Alexa Fluor 594 F(ab')_2_ fragment of goat anti-mouse IgG (Molecular Probes), both of them diluted 1∶100. Finally, cells were washed three times with PBS, coverslips were mounted in Fluoromount-G (Southern Biotech) on a microscope slide, and let at 4°C overnight. Indirect immunofluorescence was examined using a fluorescence microscope and a confocal laser-scanning microscope. To calculate the percentage of colocalization for each coverslip, the superposition of fluorescences for a minimum of 100 internalized isolated bacteria (to avoid false positives) were analyzed. These assays were performed at least two times and results were the average and standard deviation obtained from three parallel independent infections.

### Measurement of NO production by infected THP-1 cells

Supernatants were harvested after 24, 48, 72 and 96 h from cultures of PMA-treated THP-1 infected at MOI of 1∶10, 1∶1 and 10∶1. The production of NO was measured indirectly by assaying for the presence of nitrite (NO_2_^−^) using the Griess reagent. Griess reagent was added 1∶1 with supernatants. Units of NO_2_^−^ were quantified from a standard curve using dilutions of NaNO_2_ (100 nM-100 µM) as a source of nitrite, and data were normalized to NO production in non-infected THP-1 cells. After 15 min at room temperature A_540_ values were read on a spectrophotometer.

### Determination of mycobacterial susceptibility to exogenous NO

The 7H9-ADC broth was acidified to pH 5.5, with the addition of 2 N HCl, and filter sterilized. After that, approximately 5×10^3^ viable bacilli were added to tubes in 1-ml aliquots of this broth. Then, sodium nitrite was added from a sterile 1 M stock to final concentrations ranging between 0.5 µM and 10 nM. Sodium nitrite was not added to control cultures. The cultures were incubated at 37°C for 24–120 h (days 1–5), and serial dilutions were plated onto Middlebrook 7H10-OADC agar at the indicated days after infection and counted as described above. The obtained CFUs from the control cultures were considered as 100% viability.

### Statistical analyses

The statistical differences between strains were analyzed by the Student's *t* test. The levels of significance between strains were set at a *P* value of <0.05. Unless otherwise stated, all experiments were performed at least three times, and the data are given as mean values ± SD.

## Results

### Impaired replication of SO2 in human macrophages

**Figure 1:**
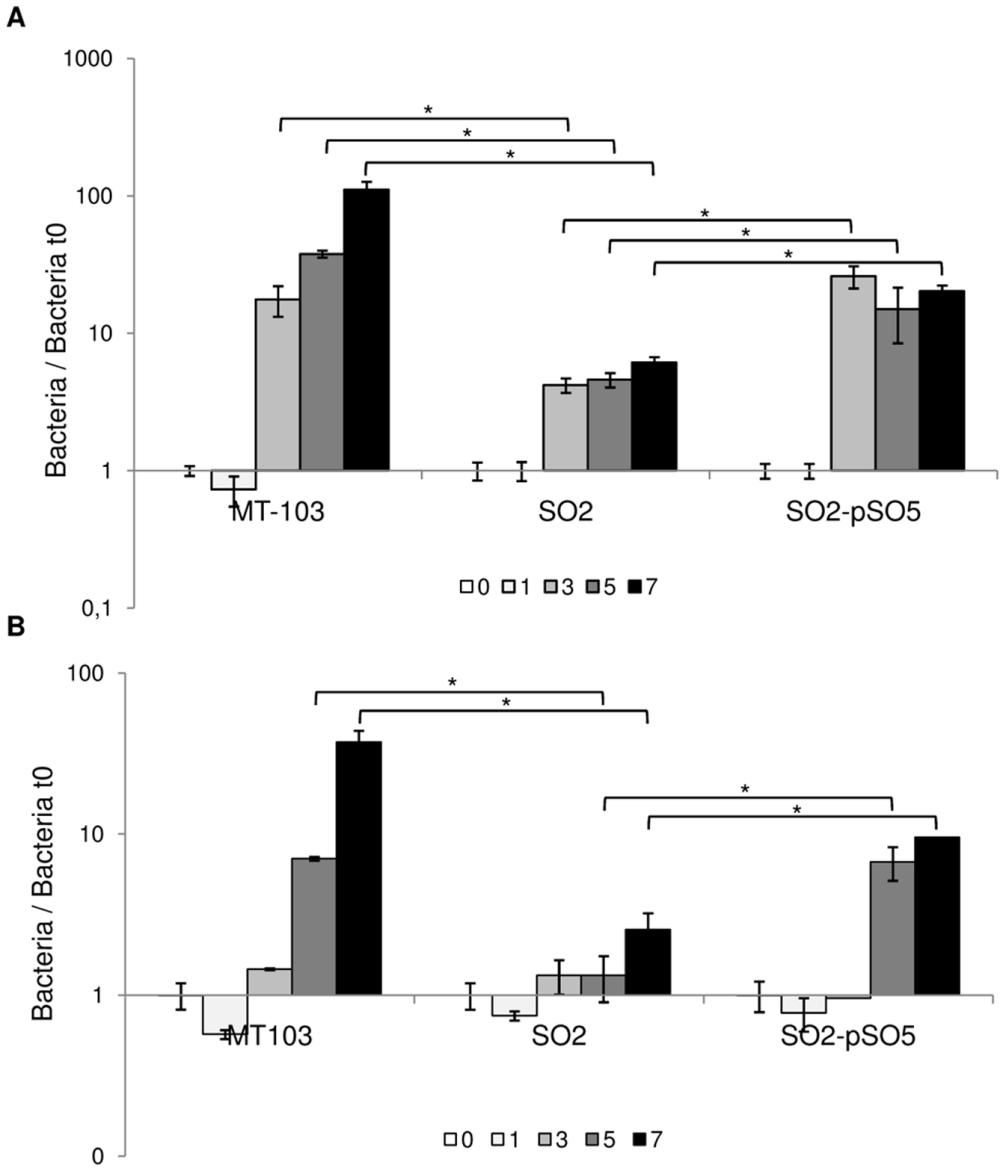
Intracellular replication within human macrophages. Data express the fold increase of cfu with respect to day 0 of infection (4 h) per well, in logarithmic scale. A) Infection of human PBMC. MOI 1∶10. B) Infection of THP-1 macrophages. MOI 1∶10. Mean values are the result of three independent experimental data ± SD, and asterisks indicate statistical significance (*P*≤0.05) using Student's t-test.

To study the intracellular replication of SO2 strain inside human macrophages, primary cells derived from PBMC and the human cell line THP-1 were infected at a MOI 1∶10 with wild-type MT103 or its attenuated *phoP*-based mutant SO2 and mycobacterial viability was studied. In both human PBMC and THP-1 derived macrophages, there was an impaired intracellular growth of SO2 when compared to MT103. At day 7 of infection of PBMC cells, MT103 had 110-fold growth increase CFU numbers, while SO2 had a lower increase of 6-fold with respect to CFU counts on day 0, indicating that SO2 replication was impaired intracellularly (about 18-fold less CFU numbers) when compared to wild-type MT103 strain (Figure 1A). The *phoP*-complemented strain SO2-pSO5 largely restored the wild type growth pattern in PBMC. Similar results were obtained in THP-1 cells, although with a smaller degree of mycobacterial growth for all strains. In this case, the numbers of intracellular MT103 bacilli increased 38-fold over the 7 days of infection (Figure 1B), whereas SO2 was characterized by a 2-fold increase in CFU as to day 0. In this cell line, the *phoP-*based strain exhibited the same degree of attenuation as in human PBMC, replicating about 18-fold less than MT103 strain at day 7 of infection. The vaccine strain *M. bovis* BCG Pasteur 1173P2 was also used in the THP-1 growth assays, exhibiting similar impaired replication as SO2 when compared to virulent MT103 strain (data not shown).

### Increased adhesion of SO2 mutant to human macrophages

As described above, human PBMC and THP-1 macrophages were infected at MOI 1∶1 (greater MOI than that in the replication assays due to the low percentage of bound bacteria). Cells were incubated during 4 h at 4°C to avoid phagocytosis, and after removing extracellular bacteria, cells were lysed to obtain only the mycobacteria bound to macrophages and analysis was made by CFU counts.

**Figure 2:**
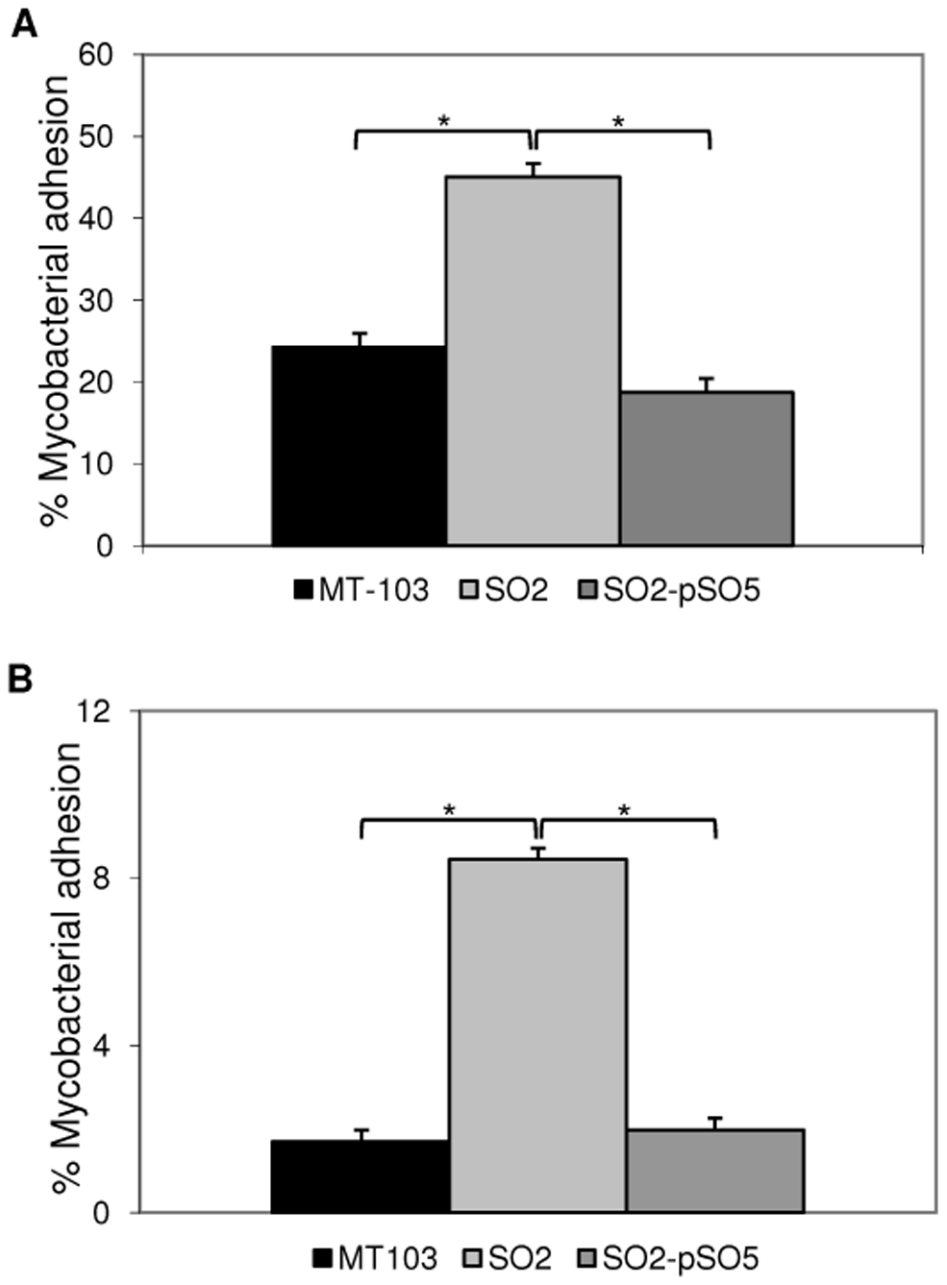
Mycobacterial adhesion to human macrophages. Data express the percentage of bound mycobacteria with respect to mycobacterial inoculum. A) Human PBMC. MOI 1∶1. B) THP-1 macrophages. MOI 1∶1. Mean values are the result of three independent experimental data ± SD, and asterisks indicate statistical significance (*P*≤0.05) using Student's t-test.

Percentage of adhesion to cells was measured as the amount of bound bacteria with respect to inoculated mycobacteria. Results showed an increase in the SO2 adhesion to human macrophages in comparison with wild-type strain MT103 (Figure 2). For PBMC, the percentage of SO2 adhesion to macrophages was approximately double that of wild-type strain (Figure 2A). For the THP-1 cell line, SO2 adhesion was up to 4-fold higher when compared to MT103 (Figure 2B).

### Alteration of SO2 trafficking within THP-1 macrophages

Due to the differences observed between SO2 and wild-type strain, both in adhesion to macrophages and intracellular growth, we sought to examine whether a significant alteration in the intracellular trafficking upon SO2 phagocytosis is produced. THP-1 cells were infected with mycobacteria and after 48 h of infection mycobacterial colocalization with cellular compartments of infected macrophages was analyzed by immunofluorescence. The MT103 strain, which evades the phagolysosome maturation, was used as negative control and heat-inactivated MT103, which follows the classical phagocytosis route, was used as positive control.

**Figure 3:**
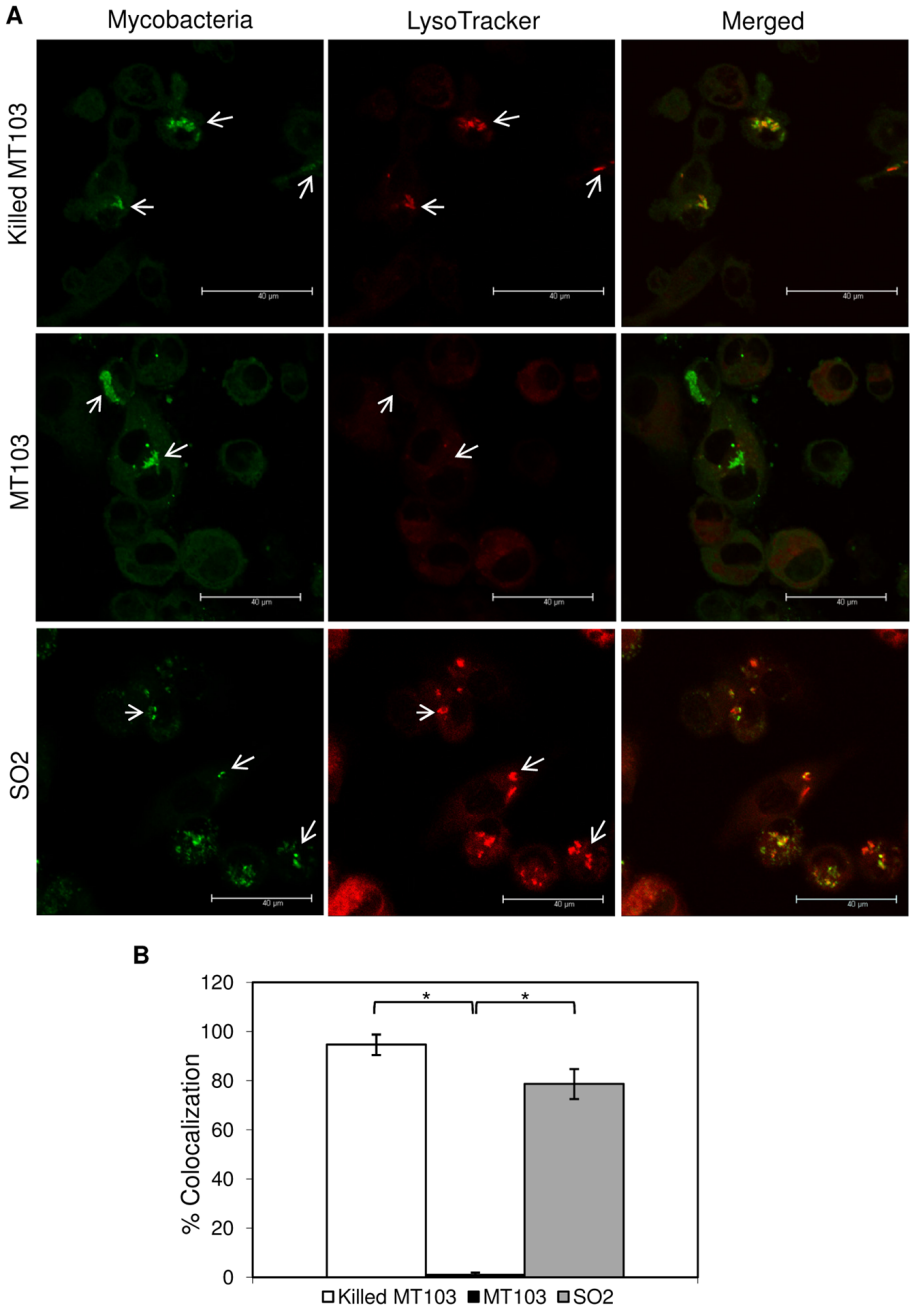
Confocal microscopy analysis of LysoTraker-stained infected macrophages. A) Representative images of THP-1 macrophages infected with live MT103, heat-killed MT103 and SO2 strains. MOI 1∶1. After 48 h of infection, cells were stained in red fluorescence with LysoTraker and mycobacteria in green fluorescence. Bars: 40 µm. Colocalization of both red and green fluorescence indicate that the mycobacteria reside in acidic compartments. B) Quantification of mycobacterial intracellular trafficking into acidified compartments. Data express the percentage of mycobacteria inside LysoTraker-compartments with respect to internalized mycobacteria into THP-1 cells. Mean values are the result of three independent experimental data ± SD, and asterisks indicate statistical significance (*P*≤0.05) using Student's t-test.

Confocal images of mycobacterial colocalization with Lysotracker showed similar colocalization of SO2 and heat-inactivated MT103 to acidic compartments of infected THP-1 cells in contrast to the wild-type strain, which hardly colocalized to these compartments (Figure 3A). For colocalization percentages, we counted isolated mycobacteria inside macrophages obtaining the same results as we observed in confocal images, a high colocalization for heat-inactivated MT103 and SO2 (approximately 95% and 79%, respectively) against 1% colocalization of MT103 (Figure 3B).

**Figure 4:**
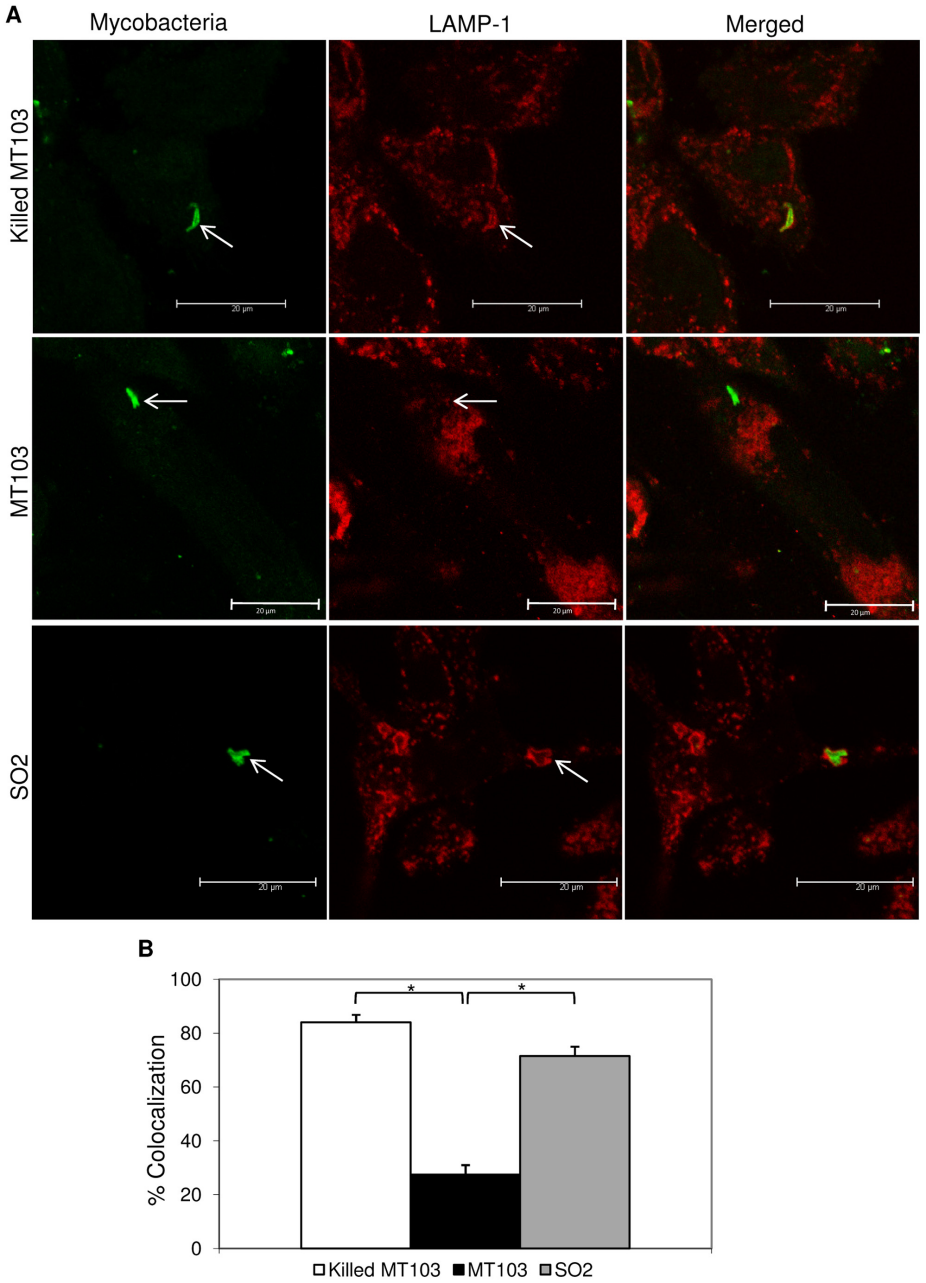
Confocal microscopy analysis of mycobacterial intracellular localization in human macrophages. A) Representative images of LAMP-1-stained THP-1 macrophages infected with live MT103, heat-killed MT103 and SO2 strains. MOI 1∶1. After 48 h of infection, LAMP-1 compartments were stained in red and mycobacteria in green. Bars: 20 µm. B) Quantification of mycobacterial intracellular trafficking into late phagosomes and phagolysosomes. Data express the percentage of mycobacteria inside LAMP-1-compartments with respect to internalized mycobacteria into THP-1 cells. Mean values are the result of three independent experimental data ± SD, and asterisks indicate statistical significance (*P*≤0.05) using Student's t-test.

**Figure 5:**
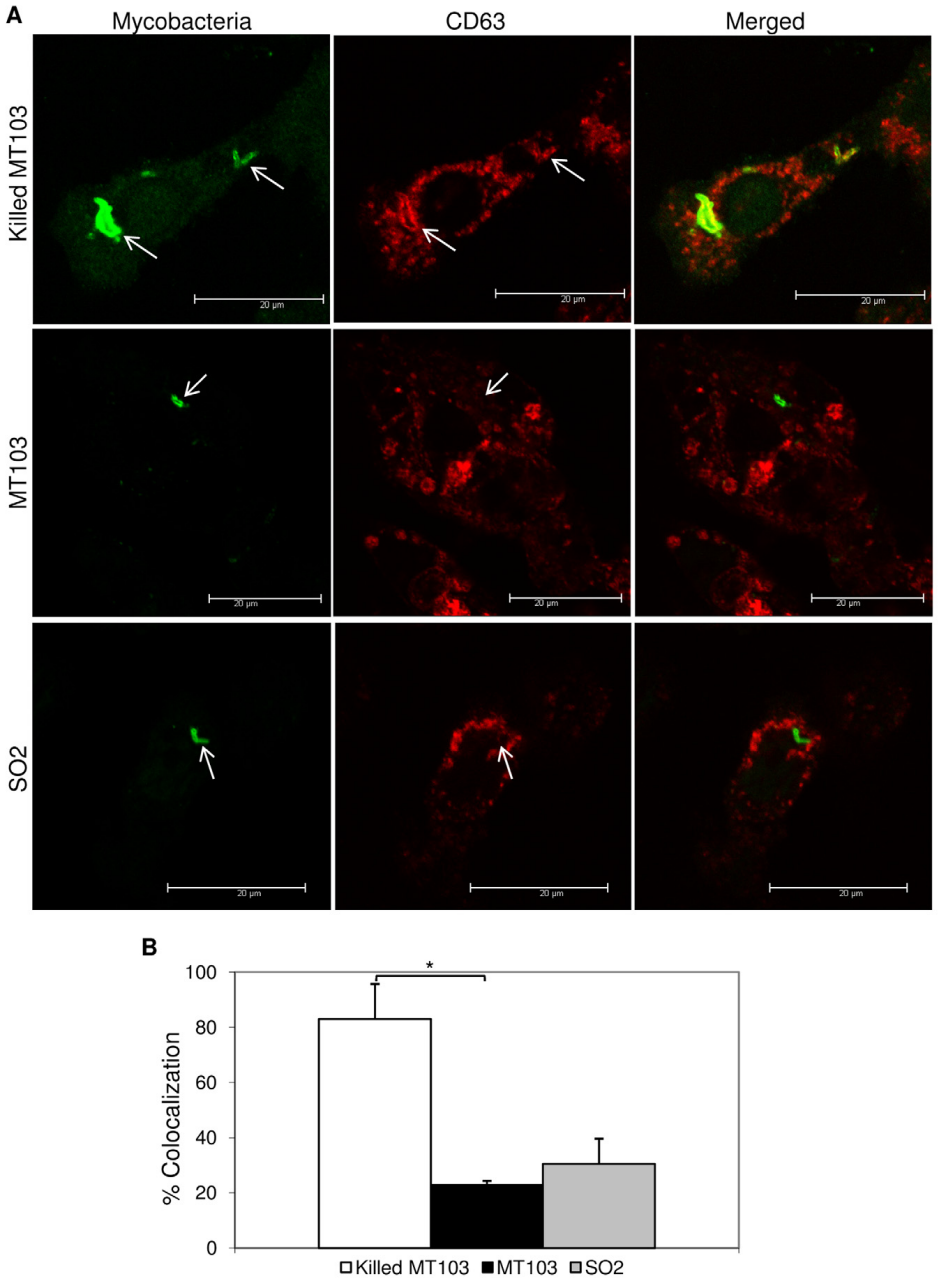
Confocal microscopy analysis of mycobacterial intracellular localization in human macrophages. A) Representative images of CD63-stained THP-1 macrophages infected with live MT103, heat-killed MT103 and SO2 strains. MOI 1∶1. After 48 h of infection, CD63 compartments were stained in red fluorescence and mycobacteria in green fluorescence. Arrows indicate colocalization. Bars: 20 µm. B) Quantification of mycobacterial intracellular trafficking into phagolysosomes. Data express the percentage of mycobacteria inside CD63-compartments with respect to internalized mycobacteria into THP-1 cells. Mean values are the result of three independent experimental data ± SD, and asterisks indicate statistical significance (*P*≤0.05) using Student's t-test.

In order to analyze in more detail the effect on phagolysosome maturation following mycobacterial infection, we studied colocolization with lysosome-associated membrane protein-1 (LAMP-1) and CD63 markers, two different lysosome-associated membrane glycoprotein characteristic of late-compartments of the phagocytic route (late endosome-phagosome and phagolysosome, respectively). Figures 4A and 5A show representative images of mycobacterial colocalization with LAMP-1 and CD63, respectively. Heat-killed MT103 and SO2 strain showed similar high levels of colocalization with LAMP-1 (70% and 80%, respectively), whereas virulent MT103 exhibited reduced colocalization (25%) (Figure 4B). However, in the case of CD63, a phagolysosome marker, both SO2 and MT103 revealed poor colocalization (35% and 20%, respectively) against the 75% colocalization observed in the infection with heat-inactivated MT103 (Figure 5B) over the period of 48 h.

### Similar NO production by infected macrophages and NO mycobacterial resistance

In addition to the phagolysosome formation, the production of nitric oxide (NO) by the infected macrophage is another mechanism of defense against Mtb in this cell type [23], [24]. In fact, inducible NO synthase expression and generation of reactive nitrogen intermediates by alveolar macrophages are increased in patients infected with Mtb [25].

To determine whether the NO mechanism is responsible for the impaired intracellular replication of SO2 strain inside human macrophages, we evaluated NO production by SO2-infected THP-1 macrophages and SO2-susceptibility to NO. NO production by THP-1 cells infected with SO2 or MT103 was measured indirectly by the presence of nitrites (NO_2_^−^) in the removed supernatants at different MOI and times of infection, using heat-inactivated MT103 as negative control. The obtained data showed similar levels of NO production by SO2 and MT103-infected macrophages in contrast to heat-inactivated MT103 (Figure S1A). These results were obtained for all MOI variations and incubation periods (data not shown), where the nitrite levels increased with the infection period and MOI in agreement with previous results described elsewhere [26]–[29]. We analyzed whether attenuation of SO2 could be characterized by greater susceptibility to NO when compared to the wild-type strain. In this context, aliquots of MT103 and SO2 were incubated at pH 5.5 in presence of increasing concentrations of NaNO_2_ (source of exogenous NO) and at days 1, 4 and 5, aliquots were plated to study the mycobacterial viability by CFU counts. The assays performed with the 0–50 µM range of NaNO_2_, which corresponds to the same NO concentration levels obtained by the infected macrophages in the previous assay of NO production, did not affect SO2 or MT103 viability even with the increase of incubation time (data not shown). However, when we used a higher range of NaNO_2_ concentrations (0.1 mM–10 mM) (Figure S1B), we observed an important decrease in mycobacterial viability for both strains with increasing NO amounts, resulting more evident at day 5 of incubation. Nevertheless, this decrease of mycobacterial viability was shown to be similar for both MT103 and SO2 strains at analyzed days, suggesting that SO2 was no more susceptible to exogenous NO than its wild-type strain.

## Discussion

The live vaccine candidate SO2 was constructed by disrupting the *phoP* gene coding for the transcription factor of the TCS PhoP/R, essential for Mtb virulence [12]. The *phoP* mutant SO2 was unable to replicate but persisted in murine macrophages and was attenuated *in vivo* in mice [12]. SO2 showed significant protective effect against TB in guinea pigs and non-human primates [14], [15] demonstrating high potential as live-attenuated vaccine candidate against TB. In the present study we addressed the intracellular replication of SO2 in human macrophages with the aim to see if the *phoP*-based mutant presents an attenuated phenotype in human macrophages.

For these assays, we selected primary macrophages from human PBMC and THP-1 cell line, which has alveolar macrophage characteristics when stimulated and differentiated with PMA [30]. In both types of macrophages SO2 demonstrated an attenuated phenotype characterized by an impaired intracellular replication with the same attenuation ratio in contrast to wild-type MT103. Due to the potential variability with PBMCs from different donors, we performed the assays with cells from various donors, obtaining consistent results for the different strains with patent attenuation for SO2. As to previous results demonstrating the inability of SO2 to replicate in murine macrophages [12], SO2 showed limited replication in the two types of human macrophages types which was also observed for BCG in the THP-1 cell line (unpublished results). Results showed similar intracellular growth pattern between both strains (data not shown), obtaining comparable degree of attenuation between BCG and SO2 in human THP-1 derived macrophages. When SO2 was complemented the replication phenotype was reverted, confirming that the *phoP* gene regulates intracellular growth in human macrophages *in vitro*, correlating with murine studies [12].

The first step in the mycobacterial infection is adhesion and mycobacterial recognition by the host cell. Recent studies have established the Mtb cell envelope as a critical determinant in Mtb–host interactions and mycobacterial virulence. In this context the lipid pattern on the mycobacterial envelope of the *phoP* mutant from MT103 was previously studied by our group [16], showing that the mutant lacks the immunomodulatory cell-wall lipids diacyltrehaloses (DAT) and polyacyltrehaloses (PAT) [16], It has been reported that deficiency in some forms of DAT and PAT affects the surface properties of *Mtb*, resulting in enhanced interaction with host cells [31]. On the other eventhough hand, these lipids are only present in the pathogenic mycobacteria, SO2 attenuation may not totally be attributed to the lack of these virulence-associated lipids, since mutants in DAT and PAT synthesis do not develop a marked reduction in virulence neither *in vitro* nor *in vivo* in mice [19], [31]–[34].

Moreover, the structure of the major non-peptidic antigen of mycobacterial cell walls, mannosylated lipoarabinomannan (ManLAM), was studied in the *phoP* mutant and it was found that there was an increase in the relative abundance of the monoacylated form of the molecule in SO2, as to wild-type MT103 suggesting that *phoP* gene could be implicated in the modulation of the acyl forms of ManLAM [35]. ManLAM plays an important role in Mtb interactions with the host through pattern recognition receptors [36]. Results from studying SO2 adhesion to human macrophages from PBMC and THP-1 cells showed significantly higher adhesion phenotype when compared to wild-type MT103. The *phoP*-complemented strain, SO2-pSO5, recovered the adhesion phenotype of the wild-type strain, suggesting that the *phoP* gene is required for this feature. The increased abundance of monoacylated ManLAMs molecules and lack of virulence-associated immunomodulatory cell wall lipids, such as DAT and PAT, (involved in mycobacterial interaction with host cells [31]), could play part in the adherence phenotype displayed by SO2. Due to this altered lipid expression profile, the change in the SO2 interaction with macrophages, characterized by a higher adhesion phenotype could be associated with a different mycobacterial association with the phagocytic receptors, leading to an alteration in the route for mycobacteria upon phagocytosis [37], [38].

Since the altered SO2 interaction with macrophages could lead to a different mycobacterial intracellular route, as well as SO2 attenuation could be due to inability to inhibit phagosome maturation characteristic of the wild-type strain, we used immunofluorescence assays to examine the intracellular trafficking of SO2 in THP-1 cells following phagocytosis. Significant differences in the intracellular trafficking between SO2 strain and wild-type MT103 were observed. Given that Mtb remains in a non-acidic mycobacterial phagosome, we first analyzed the presence of SO2 in the acidic compartments by colocalization with LysoTracker. In contrast to MT103, SO2 (like heat-inactivated MT103) failed to block the phagosome maturation indicated by high levels of colocalization to these acidic compartments. Recently the correlation between PhoP regulation and ESX-1 secretion system in intracellular mycobacteria has been described, demonstrating that ESAT-6 secretion by Mtb and specific T-cell recognition are under the control of PhoP [20], [39], [40]. The ESX-1 is required for phagosome maturation arrest [41], and the fact that SO2 has lost the capacity to export ESAT-6 in addition to the reduced expression of several other *esx-1* genes make a plausible explanation to why SO2 fails to induce phagosome maturation arrest.

In order to analyze in more detail the cell trafficking route of SO2, we used two different lysosome-associated membrane glycoproteins as organelle markers, LAMP-1 and CD63, previously described in late endosome-phagosomes and phagolysomes [38], [42]. Similar to the LysoTracker results, we observed high colocalization of SO2 with LAMP-1, late endosome-phagosome and phagolysome marker, suggesting that SO2 does not escape phagosomal maturation, in contrast to the pathogenic wild-type strain. However, when a later marker of the endocytic route was used, CD63 characteristic of the phagolysosome, SO2 mutant showed poor colocalization with this marker, displaying a persistence phenotype as described previously by our group for murine macrophages [12]. Persistence has been shown to be important for the generation of long-lasting protective immunity *in vivo* [43], supported by previous studies which demonstrated that BCG persistence results in prolonged antigen presentation and generation of memory CD8+ T cells in mice [44]. The impaired replication and the persistence phenotype of SO2 in human macrophages could be a beneficial characteristic for a vaccine candidate, being attenuated but with the potential to effectively stimulate the host immune system without causing disease. This was also described for the antigen 85A-deficient mutant of Mtb (Δ*fbpA*), a candidate vaccine with an enhanced immunogenicity profile presenting an altered intracellular trafficking behavior in macrophages, as Δ*fbpA* containing phagosomes were able to fuse with late endosomes but failed to acquire *rab*7 and CD63 markers, avoiding lysosomal fusion [45].

Apart from the phagolysosome formation, another mechanism of the innate defense against mycobacteria is the production of NO and reactive nitrogen intermediates by infected macrophages upon mycobacterial infection [23], [24], [25]. In contrast to the murine models of TB, there is a greater controversy on the role of NO in killing or limiting the growth of Mtb in humans; nevertheless, there is a growing body of evidence that NO produced by TB-infected human macrophages is also antimycobacterial against Mtb, as seen in both experimental and human TB [46], [47], [48]. Our data showed that NO production was similar for both attenuated SO2 and wild-type strains in THP-1 derived human macrophages, contrasting with previous results demonstrating that certain attenuated strains as H37Ra induced significantly higher levels of NO than their virulent counterparts H37Rv in infected PBMC cells [23]. In addition, the tolerance of mycobacteria to NO *in vitro* is strain-, dose- and time- dependent the pathogens being inherently more resistant than non-pathogenic [26]–[29]. However we demonstrate no significant differences in NO susceptibility between SO2 and wild-type MT103, discarding the NO mechanism as responsible for the SO2 attenuation (which is in agreement with previous reports from other attenuated Mtb strains [49]).

The results in this work demonstrating the attenuation, impaired replication, persistence and the different cell trafficking of SO2 in human macrophages provide a first glimpse of the behavior of this live-attenuated mutant in the human cellular model *in vitro* and are a first step to understanding the intracellular mode of action of this potential live vaccine candidate against TB.

## Supporting Information

Figure S1 NO production by infected macrophages and mycobacterial susceptibility to exogenic NO. A) NO production by infected THP-1 macrophages at day 4 (96 hours) with a MOI of 1:1. B) Mycobacterial susceptibility to exogenic NO. Data express the percentage of viable mycobacteria in the presence of 0-10 μM NaNO2, at pH 5.5, at days1 and 5. Mean values are the result of three independent experimental data ± SD, and the Student's t-test was used to determine statistical significance (P ≤ 0.05).
(9.80 MB TIF)
