# Supplementary figures and images for: Interactions of Attenuated Mycobacterium tuberculosis phoP Mutant with Human Macrophages
Source: PLoS One. 2010 Sep 24;5(9):e12978. doi: 10.1371/journal.pone.0012978 (PMC2945763; doi:10.1371/journal.pone.0012978)

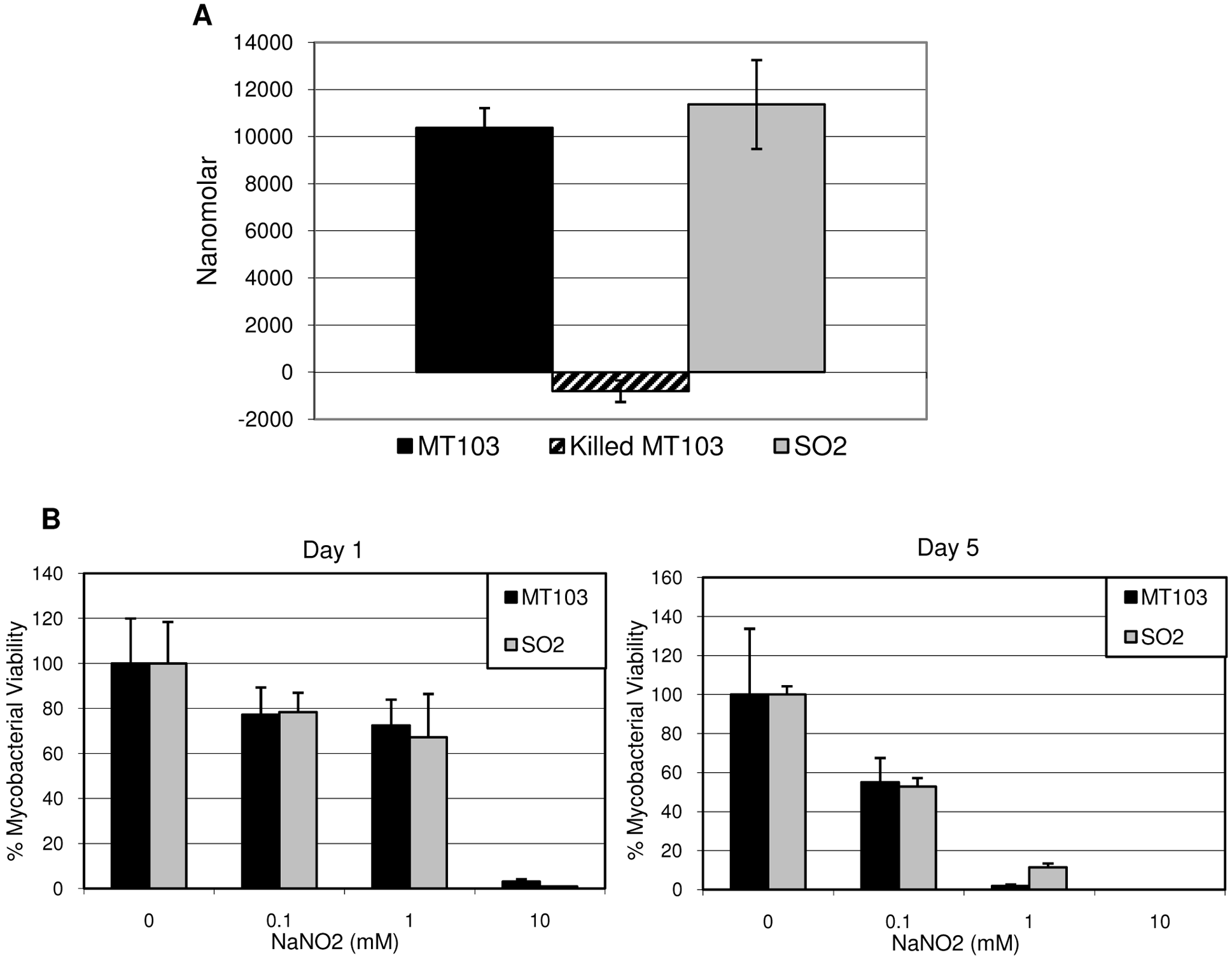

Supplement: Figure S1 — NO production by infected macrophages and mycobacterial susceptibility to exogenic NO. A) NO production by infected THP-1 macrophages at day 4 (96 hours) with a MOI of 1:1. B) Mycobacterial susceptibility to exogenic NO. Data express the percentage of viable mycobacteria in the presence of 0-10 μM NaNO2, at pH 5.5, at days1 and 5. Mean values are the result of three independent experimental data ± SD, and the Student's t-test was used to determine statistical significance (P ≤ 0.05). (9.80 MB TIF) [file pone.0012978.s001.tif]
